# Symposium on Infectious Diseases of Animals and Quarantine

**DOI:** 10.3201/eid1105.041348

**Published:** 2005-05

**Authors:** Masao Kamiya, Hong K. Ooi, Yuzaburo Oku

**Affiliations:** * Rakuno-Gakuen University, Ebetsu, Japan;; † National Chung Hsing University, Taichung, Taiwan;; ‡ Hokkaido University, Sapporo, Japan

**Keywords:** echinococcosis, avian influenza, risk

**Japan–Taiwan Symposium on Infectious Diseases of Animals and Quarantine**

Sapporo, Japan

October 20–21, 2004

The Japan–Taiwan Symposium on Infectious Diseases of Animals and Quarantine, sponsored by the Japan Interchange Association, was held October 20–21, 2004, at the Sapporo Convention Center. The symposium focused mainly on human health and food safety. Issues that were discussed included 1) infectious diseases of animals that caused economic loss, and those diseases that threaten the health of companion animals, 2) infectious diseases that are transmitted from animals to humans, 3) emerging infectious diseases that have been reported recently in world news, such as bovine spongiform encephalomyopathy and avian influenza, and 4) animal quarantine measures to prevent the spread of the aforementioned diseases. Challenges posed by infectious diseases of animals that were faced in the past, are being faced now, and will be faced in the future were highlighted in the 3 plenary lectures.

The first plenary lecture described how Japan struggled to control rabies, Japanese encephalitis, Korean hemorrhagic fever, anthrax, and other zoonoses after the Second World War (1941–1945) when poverty and poor sanitary conditions were common in Japanese. The second plenary lecture reported on the economic loss to the pig industry in Taiwan brought about by diseases such as foot and mouth disease and circovirus infection. Also described were the efforts of Taiwanese public health officials to control the epizootic diseases through the strategic use of vaccine.The third plenary lecture described our responses to a variety of risks and focused on the necessity of risk management by an ideologically mature society when it is challenged. The need to integrate diverse expertise was reiterated and examples of collaboration with mass media to solve problems were presented.

Speakers reported on the recent outbreaks of avian influenza in Yamaguchi, Oita, and Kyoto; the crisis control measures being implemented to curb the spread of the disease; and Dr. S. Y. Lin, Bureau of Animal and Plant Health Inspection and Quarantine, Taiwan, and finding and eradicating the H5N2 subtype of avian influenza virus in Taiwan. The pathogenic H5N1 avian influenza virus was found in 6 ducks in the Taiwan Sea straits. The ducks had been thrown overboard by smugglers on a boat from mainland China who were being pursued by the coast guards at Kinmen Prefecture. This occurrence illustrates that infectious diseases know no national boundary and underscores the need for a global surveillance system to prevent the spread of the virus.

One session detailed how an echinococcosis transmission was interrupted and monitored. Praziquantel-laced bait was distributed to wild foxes in Hokkaido in a mass deworming program attempting to interrupt the transmission cycle; the efficacy of the program was monitored by using the worm coporantigen detection method. This method did not disrupt the movement or eco-hierarchy of the red fox population and was sustainable in its efficacy. The drug-laced bait was produced by using marine waste products. Prevalence of echinococcosis in the red foxes in Hokkaido was reduced by using this method (*1**,**2*). This method was also successful in reducing the prevalence of the cestode infection in Zurich, Switzerland (*3*). Knowledge of being protected against infection will instill a sense of safety among the inhabitants, which in turn will lead to increased agricultural activities, as well as developing tourism industry in the region.

The control policy against echinococcosis was presented, as was the manditory reporting of infection in dogs, which became part of a revised law that became effective after October 1, 2004. This implementation is the first of its kind in the world.

Also noted was that a mutual understanding and consensus must exist among all parties concerned to effectively eradicate *Echinococcus* from red foxes. The stakeholders in this case are the inhabitants of the area, government officials, researchers, farmers, and tourist organizations.

Contemporary society is confronted with many forms of risk, including infectious disease risks and risks that threaten our food safety, that need urgent attention. This symposium brought researchers, government officials, and members of the public from Taiwan and Japan together to facilitate the bilateral regional exchange of information, with the understanding that technology that is applicable to both regions can also be extrapolated to the whole world. With the current pace of globalization, the concepts demonstrated in this symposium can serve as worldwide models.

After the symposium, the Taiwan delegation was asked for a comment on future development. The answer was that the symposium started on a bilateral basis but the discussion should be expanded to include other neighboring countries, such as Korea. Too strong a sense of nationalism may not protect national interests or the wellbeing of a nation itself.

Regarding the control of infectious diseases in animals, the symposium urged that research and development efforts tailored to specific needs be conducted. Government authorities should not ignore problems and leave them for future generations to solve. Professional organizations, such as veterinary associations, should address the problems directly; scientific societies should not limit their activities to meetings or congresses. When these aspirations take shape, the world will have changed for the better.

The abstract of this symposium is published in Japanese and English and can be obtained through a request to Kamiya Masao.
